# Positive predictive value of ultrasound in correctly identifying an inguinal hernia: a single-centered retrospective pilot study

**DOI:** 10.1186/s13244-022-01272-x

**Published:** 2022-08-13

**Authors:** Heroo Ridha, Roelof P. H. de Vries, Ingrid M. Nijholt, Saskia Abbes, Martijn F. Boomsma, Robert J. Nijveldt

**Affiliations:** 1 Department of Radiology, Isala, Zwolle, The Netherlands; 2 Department of Surgery, Isala, Zwolle, The Netherlands; 3 Department of Innovation and Science, Isala, Zwolle, The Netherlands

**Keywords:** Inguinal hernia, Ultrasound, Positive predictive value, Perioperative findings

## Abstract

**Objectives:**

To determine the clinical utility of preoperative ultrasound imaging for predicting an inguinal hernia in need of surgery. In addition, we aimed to identify factors associated with false positive (FP) ultrasound examinations.

**Methods:**

In this retrospective pilot study, we included all 175 patients who underwent inguinal hernia surgery in our hospital in 2019 and of whom a positive preoperative ultrasound examination of the groin area was available. The positive predictive value (PPV) of the ultrasound examination was determined using inguinal hernia detected during surgery (yes/no) as golden standard. To identify possible predictive factors, we compared the characteristics of patients with a FP ultrasound with patients with a true positive (TP) ultrasound.

**Results:**

PPV of ultrasound examinations to identify an inguinal hernia in need of surgery correctly was 90.9% (159/175). The patients with a FP ultrasound examination had a significantly higher body mass index (BMI) than the patients with a TP ultrasound examination (27.6 ± 4.2 vs 25.8 ± 2.3, *p* = 0.043).

**Conclusions:**

With a false positive percentage of 9.1%, there is still room for improvement of preoperative diagnostic imaging. Studies with larger cohorts are necessary to establish prediction models that have the potential to reduce FP ultrasound results.

## Key points

- Preoperative ultrasound had a PPV of 90.9% (159/175) for identifying inguinal hernia in need of surgery.
- PPV for the subgroup without a (visible) swelling was 84.6% (33/39).
- BMI was identified as most likely potential predictor of false positive ultrasounds.

## Introduction

Inguinal hernia surgery is one of the most commonly performed surgical procedures worldwide [1]. According to the national guidelines published in 2020, approximately 27,000 inguinal hernia surgeries are performed annually in the Netherlands (national guideline, 2020), of which 500 to 800 surgeries are performed in our hospital. In 95% of patients with suspected inguinal hernia, the general practitioner (GP) or surgeon can diagnose the inguinal hernia based on the clinical presentation and physical examination [1]. When the physical examination is inconclusive, ultrasonography is indicated according to both the international and Dutch inguinal hernia guidelines [1]. Typical findings of a hernia on ultrasound consist of a hernial sac containing adipose or intestinal tissue or protrusion of a herniating sac when the Valsalva maneuver is performed.

Several studies compared the role of ultrasound, magnetic resonance imaging (MRI) and/or computed tomography (CT) in the diagnosis of inguinal hernias [2–9]. In a recent systematic review (2020), Piga et al. aimed to determine which diagnostic modality is the most accurate in diagnosing inguinal hernia [2]. They concluded that ultrasound has the highest sensitivity and specificity compared to CT and/or MRI. The sensitivity and specificity of ultrasound for diagnosing inguinal hernias were 56–100% and 0–100%, respectively, whereas the sensitivity and specificity of CT scans ranged from 48 to 98% and 25 to 100%, respectively, and of MRI from 85 to 95% and 90 to 100%, respectively [2]. In spite of the broader range in sensitivity and specificity observed, on average ultrasonography performed the best.

In a systematic review by Kwee et al., the positive predictive value (PPV) of ultrasonography for inguinal hernias was evaluated [9]. In the 16 studies included, the PPV ranged from 58.8 to 100%. A pooled PPV of 86.4% (CI 95% [78.9–92.4]) was reported [9]. These findings showed that PPVs are inconsistent and vary significantly between studies. Thus, additional research is warranted. Moreover, it is currently unclear which factors are associated with false positive (FP) ultrasound findings, although operator experience has already been linked to ultrasound accuracy [2, 5, 9].

A prior clinical diagnosis of hernia may explain part of the FP diagnoses, especially when there is no evident clinical sign like palpable sac or swelling. In case of inguinal pain without swelling, diagnosing hernia may be very difficult even for a trained surgeon, GP and ultrasound technician [3].

Patients with a FP ultrasound result undergo potentially unnecessary surgery. This results in unnecessary costs, potential complications and is unlikely to solve the symptoms of the patient. Postoperative complications occur in approximately 15–28% of operated inguinal hernia patients [10].

### Aim

The primary aim of this pilot study was to investigate to what extent the diagnosis inguinal hernia on preoperative ultrasound corresponded to the perioperative findings during inguinal hernia surgery. In addition, we aimed to identify factors that may contribute to FP preoperative ultrasounds.

## Material and methods

### Study population

This pilot study concerned a retrospective cohort study. All consecutive patients (aged 18 or older) with high suspicion of an inguinal hernia, who underwent a preoperative ultrasound examination reported to be positive for inguinal hernia, and subsequently underwent hernia repair in our local inguinal hernia expertise center in 2019, were retrospectively included in this study.

All types of inguinal hernias (primary, recurrent, direct, indirect and femoral hernia) were included. All types of inguinal hernia repair, including laparoscopic (transabdominal preperitoneal (TAPP) repair and total extraperitoneal (TEP) repair) or open techniques (Lichtenstein, STOPPA), were included. Patients were excluded in case preoperative ultrasonography was performed elsewhere, when it concerned an incarcerated, strangulated inguinal hernia (medical emergency) or sports hernia, or when the ultrasound was obtained more than 6 months before surgery.

### Ultrasonography

Preoperative ultrasonography was performed on two hospital locations using two different ultrasound devices. On one location a Siemens ACUSON S2000 was used with a linear array probe 9L4 (4–9 megahertz (MHz)), 14L5 (5–14 MHz) and sometimes, in case of obese patients, the convex probe 6C2 (2–6 MHz). On the other location the Philips Epiq 7G with a linear array probe L12-3, L12-5 (5–12 MHz) or a convex probe C9-2 (2–9 MHz), C5-1 was used.

The Sectra picture archive and communication system (PACS) was used. In this PACS the images are saved in DICOM by default. Both fixed and cineloop images were made and standardized images were acquired according to protocol. All ultrasounds were generated by thoroughly trained ultrasound technicians (*n* = 33). They completed a 4-year study followed by additional training within the specific specialty. The ultrasound technicians were supervised by radiologists (*n* = 22) who read and reported the ultrasounds.

The inguinal ultrasound examination was performed according to protocol. The ultrasound technician visualized the area of the inguinal canal in supine and standing position, while performing dynamic maneuvers to detect a hernia. In case of an inguinal hernia, it was determined whether it was medial or lateral to the epigastric vessels. The contents of the hernia sac were described, measured and the reproducibility was checked. On indication, the largest lymph nodes were imaged and measured. However, there was no standardized template for documentation of these findings.

Ultrasound features of hernia included the direct visualization of a hernia sac or a positive Valsalva maneuver, which was reducible.

### Data collection

The dataset was generated using the electronic health report (EHR) search machine CTcue (CTcue B.V., Amsterdam, The Netherlands). Additional information was collected directly from the EHR.

The following patient characteristics were retrieved from clinical reports: age, gender, BMI, medical history, symptoms and the conclusion of the physical examination (swelling, size hernia, Valsalva maneuver).

Work experience of both radiologists and ultrasound technicians was collected. This included work experience in our clinic as well as previous work experience. Work experience was then categorized in groups of 5 years.

Radiology reports were reviewed for the type of transducer, ultrasound performer and interpreter, use of the Valsalva maneuver (positive or negative), size, mass and side of the hernia, epigastric veins (identified or not identified) and the conclusion (diagnosis) of the radiologist.

The following information was obtained from the surgery reports: the function of the surgeon (in training (yes/no); all trainees were supervised by an experienced surgeon), surgery technique, surgery findings and complications.

Additionally, the follow-up including the occurrence of complications (< 6 months) was collected from the EHR.

### Data analysis

Data were analyzed using SPSS version 26 (IBM, Armonk, NY, USA). The primary outcome of this pilot study was the PPV of ultrasound for the diagnosis of inguinal hernia in need of surgery. PPV was calculated for the complete study population and separately for the study population without a (visible) swelling and/or a positive Valsalva maneuver. Perioperative findings were considered the golden standard and were classified as positive or negative for inguinal hernia based on the operative reports. If intestine or adipose tissue protruded through a weakness in the abdominal wall, the inguinal or femoral canal, the patient was classified as positive. Inguinal preperitoneal lipomas were also classified as positive. All other (incidental) findings, such as an obturator hernia (unless accordingly classified by ultrasound), were classified as negative.

Herniating preperitoneal lipoma was considered as positive because they can resemble true inguinal hernias and can be treated in the same manner. Protrusion through the internal inguinal ring of extraperitoneal fat or of peritoneum with its content is both capable of producing the symptoms and signs of an inguinal hernia and should be considered equally as important as consequences of distorted regional anatomy. That is, both herniated extraperitoneal fat and herniated peritoneum and its contents are true inguinal hernias.

To identify potential predictors of FP ultrasounds, we determined whether there was a significant difference between the group patients with FP and true positive (TP) ultrasound with respect to patient age, gender, BMI, previous abdominal surgery, work experience of the ultrasound technician (years), work experience of the radiologist (years), the size of the inguinal hernia on ultrasound (cm) and the time between the preoperative ultrasound and the day of surgery. For normally distributed continuous variables (age and BMI), the independent t test was used and for continuous variables with a skewed distribution (time between ultrasound and surgery) the Mann–Whitney *U* test. The Fisher’s exact test was used for dichotomous variables (sex and previous abdominal surgery) and the Chi-square test for nominal and ordinal variables (work experience and hernia size).

Continuous variables with a normal distribution were expressed as mean ± standard deviation (SD) and continuous variables with a skewed distribution as median with interquartile range. Categorical variables were expressed as percentages.

## Results

### Study population

In 2019, 607 patients had undergone inguinal hernia surgery in our hernia repair expertise center. Of 201 patients, a preoperative ultrasound of the groin area was available. All these ultrasounds were reported to be positive for inguinal hernia. Patients with a negative ultrasound had not undergone inguinal hernia surgery. In all of the 406 operated patients who did not have an ultrasound examination prior to surgery, the diagnosis of inguinal hernia was confirmed during the surgery. Of the 201 patients with a positive ultrasound, 26 ultrasounds were excluded because they were performed earlier than 6 months prior to surgery (*n* = 17) or not performed in our hospital (*n* = 9). There were no patients with an incarcerated, strangulated inguinal hernia (medical emergency) or sports hernia. Thus, 175 patients were included in this study. There was only one patient with a lipoma. Some data were missing for the following variables: hernia size, work experience of the ultrasound technician and work experience of the radiologist.

The study population consisted of 162 men (92.6%) and 13 women (7.4%), with a mean age of 57.1 ± 14.7 years. In 129 (73.7%) cases, the groin ultrasound was requested by the GP, the surgeon requested 27 (15.4%) cases, 4 (2.3%) cases were requested by other specialists and data of 15 (8.6%) cases were missing. The linear array probe L12-5 (5–12 MHz) was used most often; 82 times (46.9%), followed by the linear array probes 9L4 which was used 53 times (30.5%) and 14L5 16 times (9.1%). The convex probes C5-1 and C9-2 were used 10 (5.5%) and 7 (4.0%) times. L12 3/4/6 have been used five times (2.9%) and 6C2 and L18-5 have both been used once (1.1%).

In 136 cases (77.7%), there was a (visible) swelling and/or a positive Valsalva maneuver during physical examination by the surgeon. The median time between the preoperative ultrasound and day of surgery was 63 (IQR 42–81) days. The most frequently used surgery technique was a laparoscopic procedure (*n* = 161), whereas the minority of patients underwent an open procedure (*n* = 14). Most (19.9%) patients had a hernia size on ultrasound between 1.0 and 1.4 cm (1.37 ± 0.92), however in 37.5% of the reports the hernia size was not described. Two patients (12.5%) with a FP result developed a postoperative complication (1 wound infection, 1 disproportional inguinal pain).

### Positive predictive value and potential predictors of false positive results

**Fig. 1:**
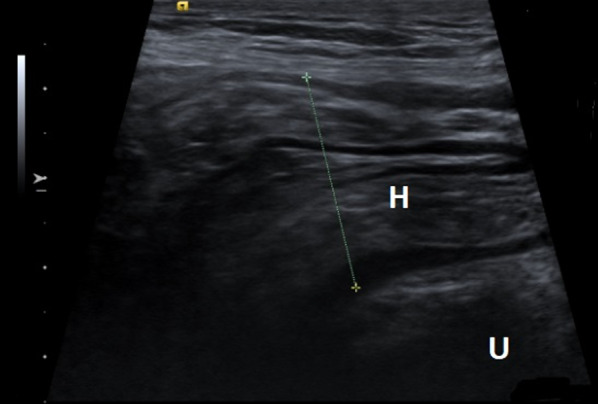
Illustration of a true positive case. Siemens ACUSON S2000 with a linear array probe 9L4 H8.00 MHz. *H* hernia, *U* urinary bladder

**Fig. 2:**
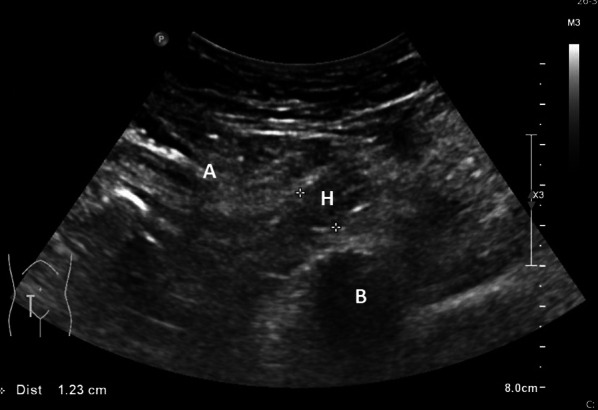
Illustration of a false positive case. Philips Epiq 7G with a convex transducer 5–1 MHz. *H* presumed hernia, *B* bone (hip), *A* abdominal wall

PPV of ultrasound for diagnosing inguinal hernia compared to perioperative findings was 90.9% (159/175). PPV for the group without a (visible) swelling was 84.6% (33/39; *p* = 0.102). Figure 1 shows a representative example of a TP case and Fig. 2 shows a representative example of a FP case.

In the TP group, most ultrasound scans were performed by technicians with 10–14 years of work experience. In the FP group, most ultrasound scans were performed by technicians with less than 5 years of work experience. However, there was no significant difference between the groups (*p* = 0.681). In both groups, most ultrasounds were read by a radiologist with more than 20 years of work experience (Table 1).

**Table 1:** Patient characteristics

Parameter	Complete study population	TP ultrasound group	FP ultrasound group	Significance (TP vs FP)
	*N* = 175	*N* = 159	*N* = 16	
Sex				0.338
Male	162 (92.6%)	148 (93.1%)	14 (87.5%)	
Female	13 (7.4%)	11 (6.9%)	2 (12.5%)	
Age in years	57.1 ± 14.7	57.7 ± 14.9	50.9 ± 10.7	0.076
BMI	26.0 ± 3.4	25.8 ± 3.3	27.6 ± 4.2	0.043
Past abdominal surgery				0.403
Yes	51 (29.1%)	48 (30.4%)	3 (17.6%)	
No	124 (70.9%)	110 (69.6%)	14 (82.4%)	
Work experience ultrasound technician				0.348
Missing	58	51	7	
Total	117	108	9	
< 5 years	37 (31.6%)	33 (30.5%)	4 (44.4%)	
5–9 years	13 (11.1%)	11 (10.2%)	2 (22.2%)	
10–14 years	44 (37.6%)	43 (39.8%)	1 (11.1%)	
15–20 years	2 (1.7%)	2 (1.9%)	0 (0.0%)	
> 20 years	21 (17.9%)	19 (17.6%)	2 (22.2%)	
Work experience radiologist				0.179
Missing	14	13	1	
Total	161	146	15	
< 5 years	20 (12.4%)	19 (13.0%)	1 (6.7%)	
5–9 years	1 (0.6%)	0 (0.0%)	1 (6.7%)	
10–14 years	24 (14.9%)	20 (13.7%)	4 (26.6%)	
15–20 years	51 (31.7%)	48 (32.9%)	3 (20.0%)	
> 20 years	65 (40.4%)	59 (40.4%)	6 (40.0%)	
Hernia size in centimeters	1.37 ± 0.92	1.39 ± 0.94	1.13 ± 0 .51	0.919
Time between surgery and ultrasound in days	63.0 (42.0–81.0)	63.0 (41.0–84.0)	60.5 (42.0–76.8)	0.686
Operation technique				
TEP	73 (41.7%)	70 (44.0%)	3 (18.8%)	
TAPP	88 (50.3%)	78 (49.1%)	10 (62.5%)	
Lichtenstein	10 (5.7%)	8 (5.0%)	2 (12.5)	
Other	4 (2.3%)	3 (1.9%)	1 (6.3%)	
Postoperative complications	30 (17.1%)	28 (17.6%)	2 (12.5%)	0.557

*TP* true positive, *FP* false positive, *N* number of patients; Significant at *p* < 0.05

There was also no significant difference between FP results of the different probe frequencies (*p* = 0.998).

In this pilot study, previously operated patients were also included. Twelve of the 175 patients had undergone a herniorrhaphy in the past. Nine of these 12 patients had undergone a Liechtenstein procedure, one patient had a TREP and for the other two patients it was unknown what kind of surgery they had undergone.

In 29 cases, the classification in hernia type was not correct; 22 lateral inguinal hernias were mislabeled as medial inguinal hernias, 5 medial inguinal hernias were mislabeled as lateral hernias and 2 femoral hernias were mislabeled as a lateral hernia.

We also registered the type of abdominal surgery the patient had undergone.

We found a significant difference between the TP group and the FP group for the variable BMI (25.8 ± 3.3 vs 27.6 ± 4.2 (*p* = 0.043)).

In the FP group, the percentage of technicians < 10 years of working experience was highest (66.6%), whereas most of the radiologists had > 15 years of working experience (60.0%) (Table 1).

## Discussion

In our study, we found a PPV of preoperative ultrasound to predict an inguinal hernia in need of surgery of 90.9%; in other words 9.1% of patients underwent surgery without having an inguinal hernia. This finding is in line with previously reported PPVs. We also identified factors that are associated with a FP ultrasound when an inguinal hernia is suspected, which, to the best of our knowledge, has not been done before in this setting.

Awareness of these factors may influence the diagnostic approach by considering alternative imaging modalities such as CT or MRI.

Although the PPV of preoperative ultrasound to diagnose inguinal hernia was investigated before, only some of these studies used surgery as golden standard. By using surgery as golden standard, a reliable PPV could be established in this retrospective study.

A higher BMI was identified as a potential predictor for FP results (*p* = 0.043). Ultrasound is difficult to perform in obese patients due to the increased distance to the target tissue [11–13]. It is more difficult to distinguish fatty tissue from material of a hernia. Moreover, the increased depth of the inguinal canal complicates identification. Compared to MRI and CT, ultrasound is the modality that is most constrained by obesity [14].

### Related literature

Accuracy of ultrasonography is in general known to be dependent on the experience of the technician [2, 5, 9]. This has however not yet been determined for inguinal hernias. We could not find a significant difference between the experience of the examiners in the FP and TP ultrasound group.

A few studies compared the accuracy of ultrasound technicians to radiologists. No studies were found specifically for ultrasound examination of the groin. Dawkins et al. [15] found an interpretation discrepancy rate of abdominal ultrasounds of 15.5% with radiologists more likely to correctly assess the ultrasounds. However, this difference was not statistically significant. A systematic review by Kwee et al. [9] registered whether the ultrasound was interpreted by a radiologist or a technician; similar results emerged from these studies.

In our hospital, the ultrasound technicians work under the strict supervision of radiologists. At the slightest doubt, the radiologist was present during the ultrasound examination. We can therefore safely assume that this had no influence on FP results.

In the past, peritoneography was the first imaging modality of choice for the diagnosis of inguinal/femoral hernia [1]. Current guidelines do not recommend this, given that significant abnormalities like a preperitoneal lipoma cannot be seen. Nowadays an MRI or CT scan is recommended when the anamnesis, physical examination and ultrasound are inconclusive [1].

### Limitations and future research

In case of a visible or palpable swelling, there is no need to perform an ultrasound unless the physical examination is inconclusive [1]. In our population, 136 of the 175 patients (77.7%) presented with a visible swelling and/or a positive Valsalva maneuver, which may have resulted in a slight overestimation of the PPV. Therefore, we also calculated the PPV of ultrasounds separately for the group without a (visible) swelling. The PPV was not significantly lower in the group without swelling than the group with a (visible) swelling (84.6% vs 90.9%, *p* = 0.102).

However, the GP or the surgeon only requested ultrasounds in case there was insufficient certainty after physical examination to make a diagnosis. The fact that the diagnosis had indeed been uncertain, can also be deduced from the finding that there were also FP outcomes (almost 10%) in the group that was considered clinically positive.

We suspected a potential relationship between previous inguinal surgery and FP results due to changed anatomy or adhesions. However, no significant difference between the two groups in changed anatomy or adhesions was found. It is important to note that no FP ultrasounds were found in the previously operated patients. Thus, it may be important in future studies to distinguish between a group with and without previous surgery.

Unfortunately, it was not possible to assess sensitivity and specificity of preoperative ultrasound since patients with a negative ultrasound were not included in our study because they often do not undergo surgery.

Due to the relatively small cohort size and a variety of surgical procedures, it was not possible to establish a model that could predict the odds on a FP ultrasound*.* For each parameter that can be evaluated in a prediction model, approximately 10–15 patients with FP results should be included.

### Recommendations

Our finding that some of the preoperative ultrasounds are FP for inguinal hernia in need of surgery, indicates the importance of detailed requests by GP’s and surgeons for ultrasound examinations. The request should at least contain the indication of the ultrasound examination, BMI of the patient, symptoms and findings of the physical examination. In this way, the radiologist has more insight in the patient’s history and factors that could potentially affect the accuracy of the ultrasound. Ideally, radiology reports should contain the following items by using a standard template: position of the patient during the ultrasound examination, use of Valsalva maneuver (positive or negative), mass and size of the inguinal hernia, epigastric veins (identified or not identified), the contents of the hernia sac, reducibility and possible limitations of the examination due to scanning conditions [16]. A more detailed radiology report is especially important in case of an inconclusive ultrasound examination.

When the anamnesis and physical examination are inconclusive and the ultrasound positive but factors associated with FP ultrasound results such as BMI are present, the surgeon may consider using additional diagnostic imaging like an MRI or CT scan as recommended by current guidelines [1]. For an accurate physical and/or ultrasound examination, it is important to examine the patient both in a standing and supine position, considering that the hernia in some cases can only be seen in a certain position or only with the Valsalva maneuver.

An MRI or CT scan with and without Valsalva maneuver is not operator dependent and may provide more certainty about the diagnosis [1]. In their systematic review, Piga et al. reported that MRI showed promising results and seems to be a better alternative than CT. However, it is important to note that not enough patients could be included in their study to draw strong conclusions [2].

Another quality-enhancing option is a multidisciplinary meeting between radiologists and surgeons to discuss patients with an inconclusive diagnosis or/and high BMI. Together they could decide on alternative treatment options such as watchful waiting, additional imaging or (diagnostic) surgery.

The use of second lectures of pictures/cineloops in abdominal ultrasonography has been investigated previously and it has been shown that this method is accurate and shows high agreement with bedside reading [17]. For diagnosing inguinal hernia, it is recommended to include a cineloop of the hernia for showing its reducibility [18].

In our hospital, cineloops are not made standard for every patient, but on indication based on the pathology. One of the reasons for this is the amount of available data storage.

We do not believe it is necessary for radiologists to read and report every ultrasound because it was previously shown that the rate of incorrect interpretations is not significantly different between the radiologists and ultrasound technicians [15]. This is, however, only true in a situation where adequate training of technicians is available and supervision by radiologists is easily accessible.

### Conclusion

We showed that preoperative ultrasound has a PPV of 90.9%. BMI was identified as most likely potential predictor of FP ultrasound results.

Patients with FP ultrasound results undergo unnecessary surgery with the risk of developing complications without solving the patient’s complaints. Moreover, these surgeries are of course also a waste of valuable time and money.

## Data Availability

The data used and analyzed are stored at Isala Hospital, Zwolle.

## Abbreviations

BMI: Body mass index
CT: Computed tomography
FP: False positive
GP: General practitioner
HER: Electronic health report
MHz: Megahertz
MRI: Magnetic resonance imaging
PPV: Positive predictive value
TAPP: Transabdominal preperitoneal repair
TEP: Total extraperitoneal repair
TP: True positive
US: Ultrasound

## References

[1] HerniaSurge Group (2018). International guidelines for groin hernia management. Hernia.

[2] Piga E, Zetner D, Andresen K, Rosenberg J (2020). Imaging modalities for inguinal hernia diagnosis: a systematic review. Hernia.

[3] Light D, Ratnasingham K, Banerjee A, Cadwallader R, Uzzaman MM, Gopinath B (2011). The role of ultrasound scan in the diagnosis of occult inguinal hernias. Int J Surg.

[4] Grant T, Neuschler E, Hartz W (2011). Groin pain in women. J Ultrasound Med.

[5] Lee RKL, Griffith JF, Ng WHA (2015). High accuracy of ultrasound in diagnosing the presence and type of groin hernia. J Clin Ultrasound.

[6] Niebuhr H, König A, Pawlak M, Sailer M, Köckerling F, Reinpold W (2017). Groin hernia diagnostics: dynamic inguinal ultrasound (DIUS). Langenbecks Arch Surg.

[7] Chmiel E, Pearson K, Mori K (2019). Over-ordering of ultrasound and pre-operative investigations for inguinal hernia repair at Northern Health: a Choosing Wisely audit. ANZ J Surg.

[8] Maisenbacher T, Kratzer W, Formentini A (2018). A value of ultrasonography in the diagnosis of inguinal hernia—a retrospective study. Ultraschall Med.

[9] Kwee RM, Kwee TC (2018). Ultrasonography in diagnosing clinically occult groin hernia: systematic review and meta-analysis. Eur Radiol.

[10] Simons MP, Aufenacker T, Bay-Nielsen M (2009). European Hernia Society guidelines on the treatment of inguinal hernia in adult patients. Hernia.

[11] Lassandro F, Iasiello F, Pizza NL, Valente T, Stefano MLMS, Grassi R, Muto R (2011). Abdominal hernias: radiological features. World J Gastrointest Endosc.

[12] Alam A, Nice C, Uberoi R (2005). The accuracy of ultrasound in the diagnosis of clinically occult groin hernias in adults. Eur Radiol.

[13] Paladini D (2009). Sonography in obese and overweight pregnant women: clinical, medicolegal and technical issues. Ultrasound Obstet Gynecol.

[14] Uppot RN, Sahani DV, Hahn PF, Gervais D, Mueller PR (2007). Impact of Obesity on Medical Imaging and image-guided intervention. AJR Am J Roentgenol.

[15] Dawkins A, George N, Ganesh H (2017). Radiologist and sonographer interpretation discrepancies for biliary sonographic findings: our experience. Ultrasound Q.

[16] European Society of Radiology (ESR) (2020). Position statement and best practice recommendations on the imaging use of ultrasound from the European Society of Radiology ultrasound subcommittee. Insights Imaging.

[17] Dormagen JB, Gaarder M, Drolsum A (2015). Standardized cine-loop documentation in abdominal ultrasound facilitates offline image interpretation. Acta Radiol.

[18] Jansen CJ, Yielder PC (2017). Evaluation of hernia of the male inguinal canal: sonographic method. J Med Radiat Sci.

